# A predictive model for classifying college students' academic performance based on visual-spatial skills

**DOI:** 10.3389/fpsyg.2024.1434015

**Published:** 2024-07-30

**Authors:** Min Ji, Jintao Le, Bolun Chen, Zhe Li

**Affiliations:** ^1^College of Marxist, Huaiyin Institute of Technology, Huaian, China; ^2^College of Educational Sciences, Yangzhou University, Yangzhou, China; ^3^Faculty of Computer and Software Engineering, Huaiyin Institute of Technology, Huaian, China; ^4^Department of Physics, University of Fribourg, Fribourg, Switzerland

**Keywords:** visual-spatial skills, academic performance, achievement prediction, classification model, neural network

## Abstract

As the application of visual-spatial skills in academic disciplines, vocational fields and daily life is becoming more and more prominent, it is of great theoretical and practical significance how to make use of big data and artificial intelligence technology to conduct research on the relationship between visual-spatial skills and students' grades. This paper explores and analyses from the perspective of artificial intelligence, combining students' visual-spatial skills and students' specific attribute characteristics to construct an expert system, which defines the prediction of academic performance as a classification problem corresponding to the five categories of excellent, good, moderate, passing, and weak, respectively, and based on which a deep neural network-based classification prediction model for students' performance is designed. The experimental results show that visual-spatial skills plays an important role in the professional learning of science and engineering students, while the classification model designed in this paper has high accuracy in the grade prediction process. This paper not only helps to fill the gaps in the current research field, but is also expected to provide scientific basis for educational practice and promote the development of the education field in a more intelligent and personalized direction.

## 1 Introduction

Students' cognitive abilities and academic performance have become one of the hot topics of research in the field of education in today's highly computerized learning environment (Esteves et al., 2023). In this context, as one of the important part of the cognitive process, visual-spatial skills have attracted widespread attention from researchers in different fields such as psychology and education.

Visual-skills are defined as a person's ability to process and understand three-dimensional spatial information, shapes, images, and spatial relationships. This skill plays an important role in students' learning, problem solving, and completion of certain tasks. As visual-spatial skills become increasingly important in disciplines, career fields, and everyday life, a theoretical understanding of how an individual's visual-spatial skills are intertwined with the demands of a discipline can help provide insight into the interplay of different skills in the cognitive process. Besides, by exploring the potential connection between visual-spatial skills and student achievement, it is expected to provide theoretical support for personalized education and optimization of teaching methods. In addition, understanding the level of students' visual-spatial skills can help educators better design curricula, provide more targeted educational services, and promote students' overall development. For example, in technical fields such as engineering, science, and medicine, where students may need to deal with and understand complex three-dimensional structures, diagrams, images, and models, visual-spatial skills can help students understand and solve mathematical problems involving graphs and spatial relationships more easily, leading to better performance in related subjects (Hegarty et al., 2020).

At present, research on the relationship between visual-spatial skills and students' performance mainly focuses on the primary and secondary school levels, and through questionnaire surveys and experimental studies, it explores how students' visual-spatial skills affect their performance in subjects such as mathematics and geography. In addition, some studies have attempted to analyze the effects of different educational environments and teaching methods on the development of students' visual-spatial skills, with a view to providing useful insights for educational reform. However, there are still some controversies and limitations in these studies. On the one hand, the results of some studies are inconsistent and may be affected by factors such as differences in study design, sample characteristics, and measurement tools. On the other hand, some studies still remain on the simple descriptions and measurements of visual-spatial skills, while more in-depth research is needed on its relationship with other cognitive skills and its mechanism of action in different disciplines.

In higher education, educational methods and teaching environments vary in their approach to the development of students' visual-spatial skills; some programs may focus more on developing students' visual-spatial skills while others may focus more on other cognitive skills. Although the importance of visual-spatial skills are increasingly recognized, there is still relatively limited research in this category on the exact relationship between it and students' grades, which may be influenced by a variety of factors (Connor and Serbin, 1985).

Currently, the education industry has been integrated with more big data, artificial intelligence and other technologies, and multidisciplinary cross-research has enabled digital education to become intelligent (Chen and Ding, 2023). In the process of college students' learning, academic performance is affected by various factors such as learning methods, motivation, subject interests, individual differences, etc. Big data analysis can guide students to achieve independent learning, inquiry learning and innovative learning, while optimizing teachers' teaching content and methods to achieve personalization and refinement of teaching. Therefore, how to use big data technology to analyze students' learning ability, learning behaviors, learning outcomes, and multiple factors related to learning in order to improve students' learning styles deserves further research (Batool et al., 2023).

In China's education student evaluation system, universities usually classify students according to their academic performance, as a way to achieve teachers' personalized guidance for students and improve their performance. Previously, the impact of visual-spatial skills on achievement was analyzed based on statistics or analyzed by specific means of intervention according to the students' learning situation, lacking a certain classification and prediction mechanism (Kahl et al., 2021). Therefore, this paper explores and analyzes from the perspective of deep learning, combining students' visual-spatial skills and students' specific attribute characteristics to construct an expert system to classify and predict research on students' performance.

In this paper, we explore and analyze from the perspective of artificial intelligence (AI) to construct an expert system for classifying and predicting students' performance by combining students' visual-spatial skills and students' specific attribute characteristics. The main contributions of this paper are as follows:

- **AI + education**: This paper proposes a student performance prediction model based on deep learning, which not only helps to provide theoretical support for the current research field, but also is expected to provide a scientific basis for educational practice, and promote the development of the education field in the direction of smarter and more personalized.
- **Diversity of data**: Multi-dimensional data from students' online classroom are collected, and visual-spatial skills are taken as an important attribute affecting students' learning ability for the design of the prediction model. The experimental results show that the idea is greatly improved in accuracy compared with the traditional prediction algorithm.
- **Data enhancement**: To address the problem of difficult sample data collection, this paper adds the operation of data enhancement in the preprocessing process of the model, which makes the model learn the features and laws of the data in a better way by introducing more data samples and diversities, so as to improve the performance of the model on various tasks.

## 2 Related work

With the deepening of educational research, the relationship between visual-spatial skills and students' grades has also attracted extensive interest from scholars. Research on the relationship between the two has made some progress both internationally and domestically. At present, the methods of predicting learners' grades can be mainly categorized into the following two groups: (a) statistical-based methods, such as cluster analysis, regression analysis, etc.; (b) machine learning-based methods, such as genetic algorithms and neural networks.

### 2.1 Statistics-based methods

Fanari et al. (2019) investigated the effect of visual-spatial ability and math grades of children at different time intervals by using models such as Bayesian regression, and the results of the experiment showed that the importance of visual-spatial ability on math grades tended to decay as the age increased. Lufler et al. initiated a study on the effect of visual-spatial ability on medical students' overall performance in an anatomy course using logistic regression analysis and student modeling. The results of the study showed that students' visual-spatial ability was an effective predictor of performance in medical gross anatomy, while early intervention for students with low visual-spatial ability could improve their later academic performance (Lufler et al., 2012). Obando et al. investigated whether visual-spatial ability could lead to changes in the teaching of medical anatomy and whether it could predict academic performance in the subject. The results of the study showed that the delayed memory of the instrument predicts academic performance, while anatomical learning improves students' visual spatial functional performance (Caldichoury-Obando et al., 2022).

Shi et al. used structural equation modeling to analyze the correlation between five cognitive abilities, namely, memory ability, representation, information processing, logical reasoning, and thought conversion, and academic grades using students' self-monitoring as a moderating variable. The results of the study showed that there was a positive correlation between cognitive ability and academic grades. Meanwhile, self-monitoring plays an important role in moderating the effect of cognitive ability on academic grades (Shi and Qu, 2022). Reyes et al. studied the impact of learning strategies, learning habits, and learning attitudes on grades through statistical analysis. Due to the ordinal nature of the data and the lack of multivariate normality, they used weighted least squares means and variance correction to design a structural equation modeling (Reyes et al., 2023). Rohde and Thompson analyzed the relationship between cognitive abilities such as visual-spatial working memory, information processing speed, and academic achievement of students from first to eleventh grade through structural equation modeling. The results of the study showed that the contribution of cognitive characteristics to individual differences in academic success declined from elementary school to the completion of general education (Rohde and Thompson, 2007).

Liu et al. predicted the future academic performance of recruited Chinese children by measuring their visual-spatial, arithmetic and reading abilities. Analyzing the results using SPSS, they found that there was a significant correlation between visual-spatial, arithmetic, reading abilities and academic grades. In addition, visual-spatial skills can indirectly affect students' academic grades through arithmetic and reading abilities (Liu et al., 2021). Pluck used correlational and cross-sectional methods to assess the visual perceptual ability of industrial engineering students and social science students. The results found that visual perception abilities, especially size judgement abilities, were significantly and positively correlated with industrial engineering students' grades, while students' usual grades were a significant predictor of academic performance (Pluck, 2023). Tian et al. investigated the relationship between performance on various parallel visual tasks and academic achievement through Spearman's correlation analysis and Mann-Whitney *U*-test. The results of the study showed that school-age children's ability to process specific parallel visual tasks was related to Chinese language achievement, and that the risk of this factor on learning could be reduced by providing appropriate assistance to students affected by visual problems (Tian et al., 2023).

After analyzing a large body of literature, Xie et al. (2020) found that the relationship between spatial and mathematical abilities is not simply linear, and that their relationship is not moderated by factors such as spatial ability, age, or developmental status. Krajewski and Schneider (2009) showed that visual-spatial working memory and phonological awareness influenced the development of mathematical ability from an early age after a 3-year longitudinal study of students. However, Hawes and Ansari (2020) investigated the underlying mechanisms of the relationship between spatial and mathematical abilities and concluded that there is no conclusive answer to the question of whether spatial training is an effective means of improving numerical thinking, and that differences in training methodology are the main factor in the differing findings.

The majority of methods based on statistical analyses are focused on the collection of student data, followed by an in-depth study of the attentional mechanisms and cognitive processes that students use to process spatial information over time, and then ultimately correlated using statistical methods. Such methods lack some predictive mechanisms, and they do not have a conclusive way of characterizing the importance of which data are collected and how they are processed.

### 2.2 Machine learning-based methods

Pallathadka et al. (2023) conducted research on machine learning methods such as Nave Bayes, ID3, C4.5 and SVM. The experimental results showed that SVM performed best in predicting student grades. Badal and Sungkur (2023) also used machine learning techniques to predict students' grades and participation. Through quantitative methods of analyzing and processing student data, it was proved that the random forest classifier was superior to other classifiers. Pandey and Sharma (2013) selected eight important attributes among 18 attributes affecting students' performance by calculating the information gain rate of each attribute feature, on the basis of which they constructed a decision tree to predict students' performance. Xu et al. (2020) collected data on students' learning behaviors such as study hours and scores for answering subjective questions by creating a blended learning scenario, and then used a multiple regression model to predict their academic performance. Son and Fujita (2019) proposed a Multi Adaptive Neuro-Fuzzy Inference System with Representative Sets (MANFIS-S) to predict the future performance of students once they enter college. Keser and Aghalarova (2022) proposed a hybrid integration algorithm of Boosting and Stacking to predict students' academic performance and achieved better prediction results.

Oyedeji et al. (2020) used supervised learning based linear regression, deep learning based linear regression, and neural networks for prediction of students' academic grades after collecting their personal attributes including age, demographic distribution, family background and attitude toward learning, respectively. Vijayalakshmi and Venkatachalapathy used Decision Tree (C5.0), Plain Bayes, Random Forest, Support Vector Machine, K Nearest Neighbors and Deep Neural Networks (DNN) to predict students' academic ability based on their demographic, academic background, and some of the behavioral characteristics. The experimental results showed that deep neural networks had the highest accuracy rate among the six algorithms (Vijayalakshmi and Venkatachalapathy, 2019). Based on the attributes of students' school, gender, age, and number of absences from class, Li et al. proposed a prediction model for students' performance based on a two-way attention mechanism, which not only treats the degree of influence of these factors on performance differently, but also takes into account the individual variability of students. The results show that the constructed model can predict students' performance more accurately and has good interpretability (Li et al., 2020). Giannakas et al. (2021) proposed a binary classification deep neural network with two hidden layers to predict the performance of team members during collaboration on a project by collecting data generated by the team during collaboration.

Although some progress has been made in the prediction of grades through machine learning methods, theses methods only consider some attribute features related to the team's project in their model design, and do not take into account the influence of factors such as the inherent visual-spatial skills of the team members themselves on the prediction of grades. Therefore, how to select valuable input features and how these features can contribute to the prediction of machine learning models are also the focus of the next research on this type of methods.

## 3 Proposed method

While academic grades are influenced by a variety of factors such as learning methods, motivation, subject interests and individual differences, visual-spatial skills play a crucial role in academic grades. Therefore, research on this issue needs to consider a variety of factors in order to fully understand their influence on each other.

### 3.1 Student performance prediction algorithm framework based on deep neural network

We rank the students' grades from highest to lowest and define them as five levels of excellent, good, moderate, passing and weak according to 15, 25, 40, 15, and 5%, respectively. Therefore, this paper defines the prediction of academic performance as a classification problem with five categories, with the aim of assigning a category label to each student to be predicted, corresponding to the five grades of excellent, good, moderate, passing and weak. In this paper, a deep neural network-based classification prediction model for student performance is proposed, taking into account students' visual-spatial skills. The model employs a deep neural network framework with powerful feature learning capabilities to optimize data features using three fully connected layers to classify student grades through the use of Support Vector Machines (SVM). The model is proposed to provide teachers with additional suggestions for personalized teaching and the detailed steps are shown in Table 1.

**Table 1:** A deep neural network based algorithmic framework for student performance prediction.

**Algorithm 1 GNN-S Algorithm**.
**Input**: Sample attribute dataset *X*, sample label dataset *Y*
**Output**: The prediction result *Predict*_*label*
**Begin**
1. A preprocessing operation is performed based on the input student data to extract the attribute dataset *X* of the samples and the corresponding label dataset *Y* of the samples;
2. Normalization of the sample data *X* and data augmentation of the data to obtain the sample data matrix *X*′;
3. The matrix *X*′ is partitioned into data sets to obtain the training set *X*^*train*^ and test set *X*^*test*^;
4. The training set *X*^*train*^ is fed into a deep neural network for predictive classification and compared to *Y*^*train*^, and this network model is iteratively optimized;
5. The test set *X*^*test*^ is input to the trained DNN for feature extraction;
6. The information extracted from the features is fed into SVM to produce predictions *Y*^*predic*^ corresponding to the test set *Y*^*test*^;
7. Evaluation of forecast results using evaluation indicators.
**End**

### 3.2 Detailed steps of the algorithm

#### 3.2.1 Data initialization

**Figure 1:**
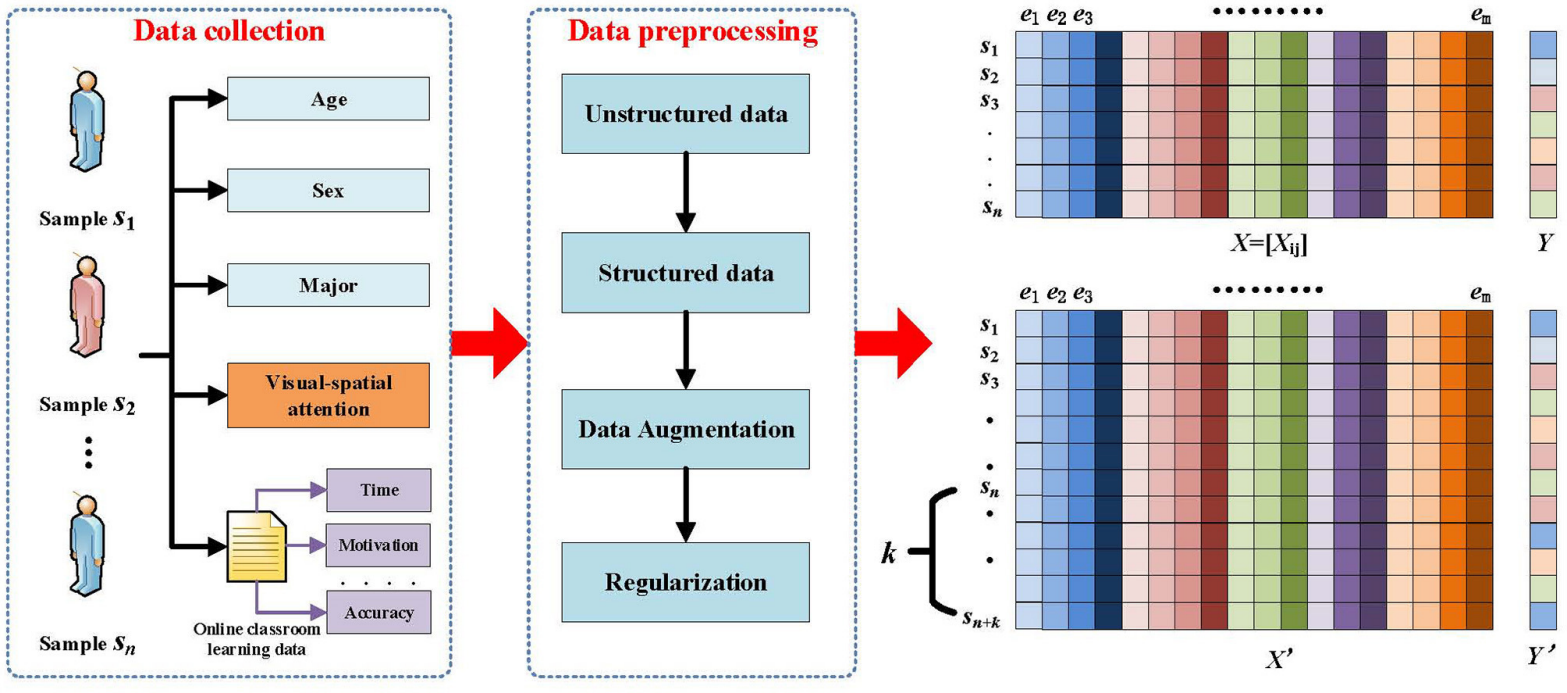
Mathematical model of student performance classification problem.

During the design process of the student performance prediction algorithm, we need to deal with multidimensional student sample data, such as age, gender, major, visual-spatial skills, as well as data related to students' classroom performance collected from the online classroom, such as video learning hours, number of questions asked, instructor evaluations, and class assignments. For the collected sample data, we can use the following data structure for sample data storage. We define *A* = {*S, E*} as the sample data network of students, where *S* = {*S*_*i*_} is the set of students in the sample and *E* = {*e*_*i*_} is the set of attribute features. We set *X* = [_*x*_*ij*_]*n*×*m*_ to be the feature matrix consisting of the values of attribute *j* of sample *i*, and *Y* = [_*y*_*i*_]*n*×1_ to be the grade category label corresponding to each sample. Thus, student grade prediction is to assign a category label *y*_*i*_∈{1, 2, 3, 4, 5} to each student feature vector *x*_*i*_ = {*x*_*i*1_, *x*_*i*2_, ..., *x*_*im*_} to be predicted, corresponding to the five grades of excellent, good, moderate, passing, and weak, respectively. A schematic of the mathematical model for the student achievement classification problem is given in Figure 1.

In the process of model training using deep learning techniques, it is generally required that the number of samples should be sufficient, the more the number of samples, the better the effect of the trained model, and the stronger the generalization ability of the model. However, in practice, the number of samples is insufficient or the sample nature is not good enough, which requires data enhancement of the samples to improve the quality of the samples. Therefore, we use Gaussian noise technique on the original dataset to generate new but still real and reliable training samples to improve the generalization ability of the model, and to improve the robustness of the model by increasing the noise data. The method utilizes a specific Gaussian distribution of noise with set parameters of mean μ and standard deviation σ to generate noisy data similar to the original data with the specific probability density function defined as shown in Equation 1:


(1) $$\begin{array}{l} pdf\left(t\right)=\frac{1}{\sqrt{2\pi \sigma^{2}}}e^{-\frac{{\left(t-\mu \right)}^{2}}{2\sigma^{2}}} \end{array}$$

Where, *t* represents the random variable, μ is the mean, and σ is the standard deviation. This step first copies the original data *X* and labels *Y* into new variables *X*′ and *Y*′ to ensure that the augmented data does not affect the original data; then, in a set number of augmentation rounds, a specific Gaussian noise distribution is added to each row of *X* for each round, so that a new data point with noise is generated for each original data point; finally, the generated data is added to the array *X* using data stacking; similarly, *Y*′ is correspondingly is grown by vertically stacking the same labels to match the amount of enhanced data. This approach expands the dataset while keeping the labels the same, compensates for the lack of dataset, and increases the generalization ability of the model.

Next, we perform *Z*-score standardization on each column of the sample data so that the processed data conforms to the standard normal distribution, i.e., the mean is 0 and the standard deviation is 1. Data standardization counteracts the error caused by the different feature scales of each attribute, and standardization is a linear transformation, all of which are compressed according to the proportion of a feature attribute in the sample data, followed by a panning operation. Data normalization not only does not change the numerical order of the original data, but also improves the performance of the data. The specific normalization function is defined as shown in Equation 2.


(2) $$\begin{array}{l} X^{\prime }=\frac{X-\mu }{\sigma } \end{array}$$

The μ in the formula is the mean of each column of attribute data and σ is the standard deviation of each column of attribute data.

In order to test the accuracy of the algorithm, the set of sample data *X*′ with known labels needs to be divided into two parts: The training set $X^{train}=\left\{x_{i}|i\in \left\{1,...,n+k\right\}\right\}$ and the test set $X^{test}=\left\{x_{j}|j\in \left\{1,...,n+k\right\}\right\}$. Only the information in the test set can be used in calculating the labels of the predicted samples, obviously, *X*′ = *X*^*train*^∪*X*^*test*^, and *X*^*train*^∩*X*^*test*^ = ϕ, where each experimental result is the average value obtained by predicting and evaluating the training set (containing 60% of the number of samples) and the test set (containing 40% of the number of samples), which are formed by randomly dividing the original data set for 100 times.

#### 3.2.2 Deep neural network-based feature extraction

**Figure 2:**
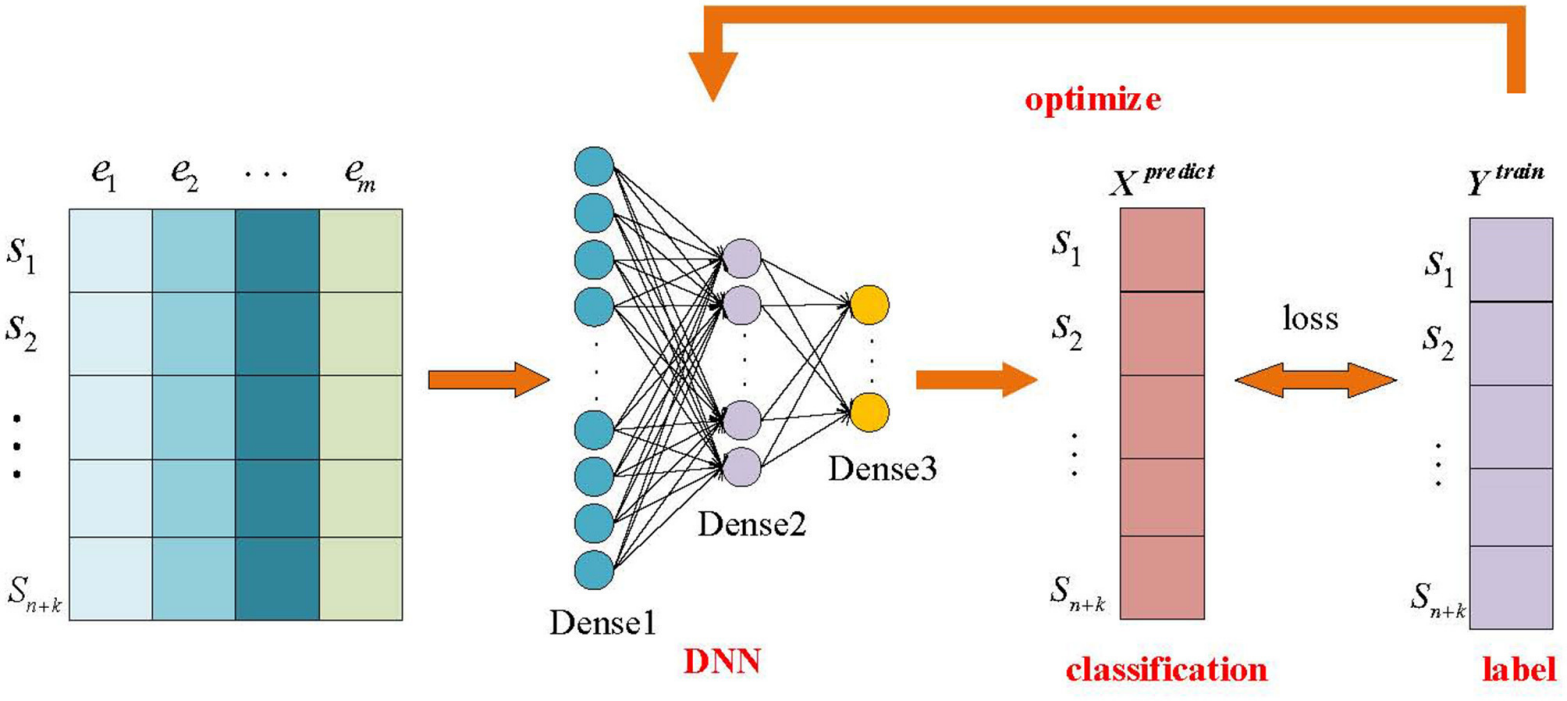
Processing mechanism based on deep neural networks.

For the preprocessed data *X*^*train*^ and *Y*^*train*^, we input them into the DNN to get the predicted labels and calculate the loss function based on the predicted labels *X*^*predict*^ and the true labels *Y*^*train*^, and use this to optimize the model DNN, the specific process is shown in Figure 2.

In this step, for each sample of input data *x*, the value of the *j*th cell in layer *i* is noted as ${\hat{h}}_{j}^{i}\left(v\right)$, where *i* = 0 denotes the input layer and *i* = *l*+1 denotes the output layer, and we denote the size of the layer as $\left|{\hat{h}}^{i}\left(v\right)\right|$. The default activation level is determined by the internal bias $b_{j}^{i}$ of the cell. The set of weights between ${\hat{h}}_{j}^{i-1}\left(v\right)$ in layer *i*−1 and ${\hat{h}}_{j}^{i}\left(v\right)$ in layer *i* determines the activation of ${\hat{h}}_{j}^{i}\left(v\right)$.

The information aggregation process of DNN is shown in Equation 3:


(3) $$\begin{array}{l} \;{\hat{h}}_{j}^{i}(v)=sigm\left(a_{j}^{i}(v)\right),\text{ where }a_{j}^{i}(v)=b_{j}^{i}+\\ \sum_{k}w_{jk}^{i}{\hat{h}}_{k}^{i-1}(v)\forall i\in \{1,\ldots ,l\},\text{ with }{\hat{h}}^{0}(v)=v \end{array}$$

Where *sigm* (·) is the *sigmoid* function, defined as follows: $sigm\;\left(a\right)=\frac{1}{1+e^{-a}}$. Given the last hidden layer, the output layer is computed as shown in Equation 4:


(4) $$o(v)={\hat{h}}^{l+1}(v)=f\left(a^{l+1}(v)\right),\text{ where }a^{\text{l+1}}(v)\text{ = }b^{\text{l+1}}\text{+ }w^{\text{l+1}}{\hat{h}}^{\text{l}}(v)$$

Where the activation function *f* (·) depends on the task that the network must perform. Typically, it will be the constant and *softmax* function for regression problems, where *k* refers to the number of classes. The specific steps are shown in Equation 5.


(5) $$f_{j}(a)=softmax_{j}(a)=\frac{e^{a_{i}}}{\sum_{k=1}^{Z}e^{a_{k}}}$$

Next, a deep neural network was used to carry out by constructing three fully connected layers, the first layer included 64 neurons to initially extract data features from the raw data; the second layer was reduced to 32 neurons in order to further integrate the features to extract the information; and the third feature layer, which contained 16 neurons, was further optimized to summarize the outputs of the first two layers and to generate a feature set that is more useful for classification. The training set *X*^*train*^ is preclassified after the deep neural network and the training set labels *Y*^*train*^ are calculated using the *MSE* loss function to derive the loss function *loss*, whose specific formula is shown in Equation 6:


(6) $$loss=\frac{\sum_{i=1}^{n}{\left(Y_{i}^{train}-X_{i}^{predict}\right)}^{2}}{n}$$

Where *n* is the number of samples, $X_{i}^{predict}$ is the model's predicted label for the *i*th sample in the training set, and $Y_{i}^{train}$ is the true label for the *i*-th sample in the training set.

Next, starting from the output layer, we first compute the gradient of the loss function *loss* with respect to the output of the output layer, and then back propagate the gradient using the chain rule. Immediately after that, the gradient computed by back propagation is used to update the network parameters in order to reduce the value of the loss function and thus perform the optimization of the model. In this paper, this process is implemented by the optimization algorithm *adam*, which enables the model to gradually approach the optimal solution to obtain a trained deep neural network. Finally, the test set *X*^*test*^ is input to the trained deep neural network to the extracted features.

#### 3.2.3 Constructing a classification prediction model using SVM

**Figure 3:**
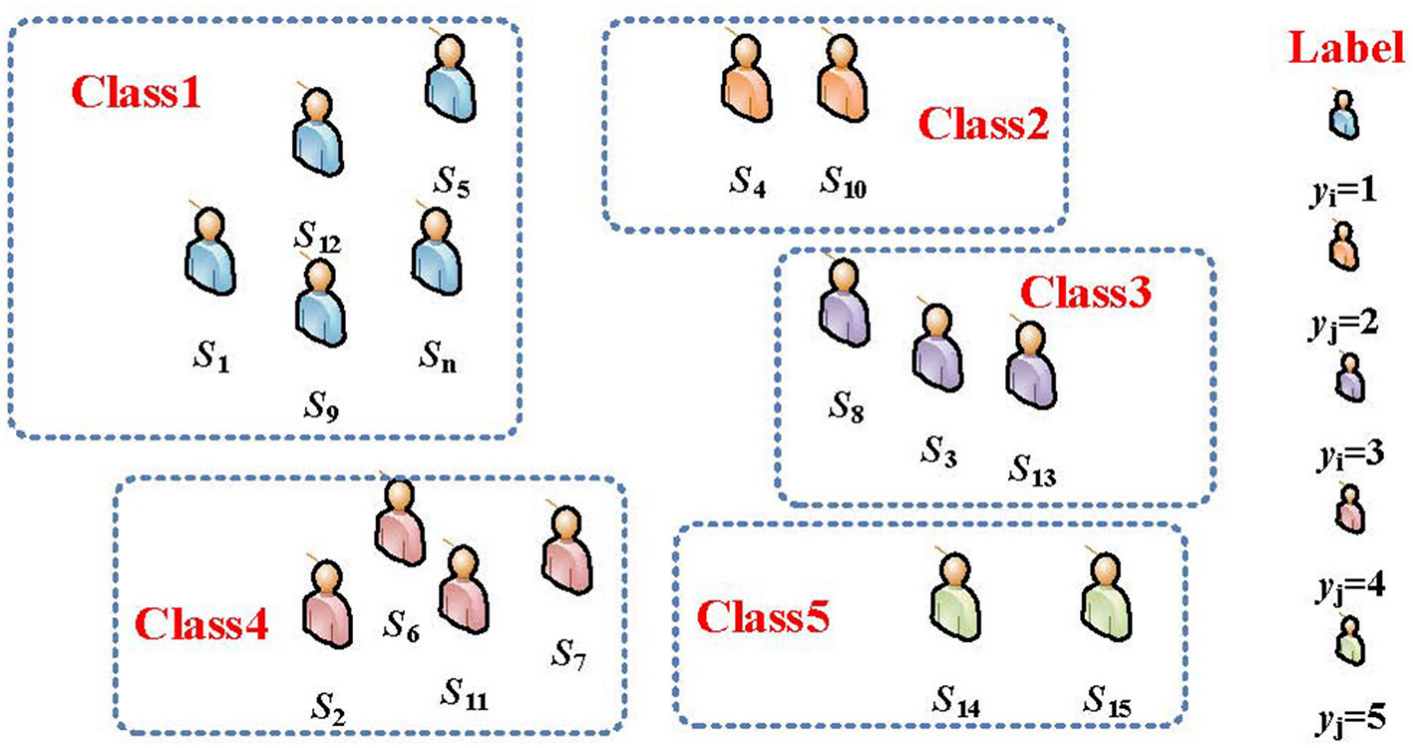
Schematic diagram of the algorithm for classifying student performance.

In the last step of student performance prediction, we propose to transform it into a classification problem. The basic idea of the classification algorithm is to find an optimal division hyperplane in the feature space based on the training set *X*^*train*^ to separate the five categories of samples corresponding to the performance. That is, the original inseparable data is mapped to a new space, and the conversion is classified in the new space. In the classification problem of student performance prediction, we use result *X* of the DNN output in the previous step as the input part of the classification algorithm SVM to derive the training model. Finally, the prediction result *y*_*i*_∈{1, 2, 3, 4, 5} corresponding to the test set can be obtained based on the training model. The schematic diagram of student grades classification is shown in Figure 3.

#### 3.2.4 Evaluation indicators of the model

After the prediction model is designed, we need to evaluate its effectiveness, and the common metrics used to measure the accuracy of the algorithm are *Accuracy*, *Precision*, *Recall*, and *F*1_*Score*. We first introduce the following four concepts:

- True positive (*TP*) means that the predictive model correctly predicts a positive category sample as a positive category.
- True negative (*TN*) means that the predictive model correctly predicts a negative category sample as a negative category.
- False positive (*FP*) means that the predictive model incorrectly predicts a negative category sample as a positive category.
- False negative (*FN*) means that the predictive model incorrectly predicts a positive category sample as a negative category.

*Accuracy* is the ratio of outcomes that our model predicts correctly, defined as shown in Equation 7:

(7) $$\begin{array}{l} Accuracy=\frac{TP+TN}{TP+TN+FP+FN} \end{array}$$

*Precision* is the proportion of samples identified as positive categories that are indeed positive, defined as shown in Equation 8:

(8) $$\begin{array}{l} Precision=\frac{TP}{TP+FP} \end{array}$$

*Recall* is the proportion of samples that are correctly identified as positive categories out of all positive category samples, defined as shown in Equation 9:

(9) $$\begin{array}{l} Recall=\frac{TP}{TP+FN} \end{array}$$

*F*1_*Score* combines the results of *Precision* and *Recall* and is a weighted reconciliation average of *Precision* and *Recall*. When the *F*1_*Score* is high then it can indicate that the test method is more effective. The definition is shown in Equation 10:

(10) $$\begin{array}{l} F1\text{\_}Score=\frac{2\times Precision}{Precision+Recall}=\frac{2\times TP}{2\times TP+FP+FN} \end{array}$$

For the final model, we propose to validate the performance of the model using the four metrics as above.

## 4 Result and discussion

### 4.1 Participants

In this research, a total of 29 students from a class of computer science majors in our university were recruited as a sample for one year of follow-up data collection. Their median age was 24 years (range = 23−25), with 16 males (55.17%) and 13 females (44.83%). All participants were screened for sensory impairments that could affect performance, and all had normal vision or corrected vision (e.g., glasses). Data augmentation can improve the performance and effectiveness of deep learning models; by introducing more data samples and diversity, the model can better learn the features and patterns of the data, thus improving the model's performance on various tasks. Therefore, we used Gaussian noise data augmentation on the basis of the original data to expand the data volume by 101 times from the original 29 entries, which maintains the authenticity of the data and makes up for the lack of the original data volume.

In this paper, the data collected for student attribute characteristics are mainly divided into three categories, which contain students' basic information including students' gender, visual-spatial skills data, and online classroom learning data, respectively. The specific division results are shown in Table 2.

**Table 2:** Category of each attribute.

**Attribute types**	**The specific attributes**
Basic information	Age, sex
Visual-spatial skills	Spatial rotation ability, observation, visual sensitivity, perception of scale and size, detail discrimination
Online classroom learning data	Number of absences, learning discussions, outstanding speeches, number of video learning, etc.

**Figure 4:**
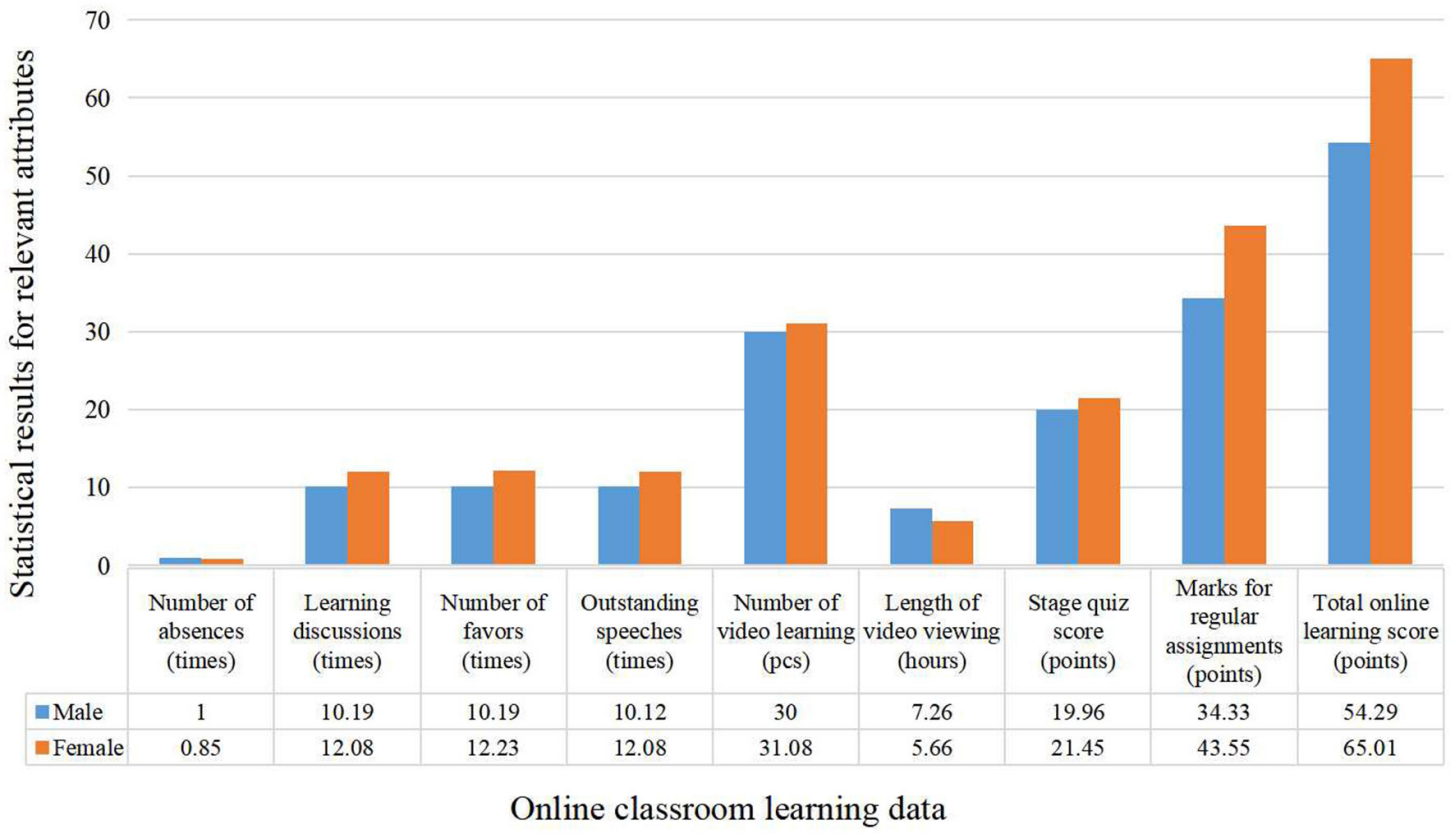
Statistical results of online classroom learning data.

Among them, the statistical results of the learning data in the online classroom are shown in Figure 4.

From the data related to gender differences in the online classroom, it is clear that female students outperform their male counterparts in several important dimensions. Specifically, in terms of the manifestation of learning attitudes, by looking at the average number of absences, it can be found that female students present a lower rate of absenteeism than their male counterparts, which is indicative of a higher degree of commitment to learning among female students. In terms of classroom interaction, female students outperform male students in terms of the number of discussions and the number of presentations rated as outstanding, which reflects the fact that female students are more active than their male counterparts in terms of participation in the classroom. Although the frequency and quantity of videos watched by female students is slightly higher than that of male students, male students watch videos for a significantly longer period of time than female students. This statistic suggests that male students differ from female students in the utilization of learning resources. However, on quiz and assignment grades, which are direct measures of online classroom performance, female students' average grades were significantly higher than those of male students, which is further evidence of female excellence in classroom outcomes. These findings reveal how gender differences manifest themselves in online learning environments and may refer to the fact that these data will play an important role in predicting student performance at a later stage.

### 4.2 Data collection and analysis

#### 4.2.1 Tests of visual-spatial skills

**Figure 5:**
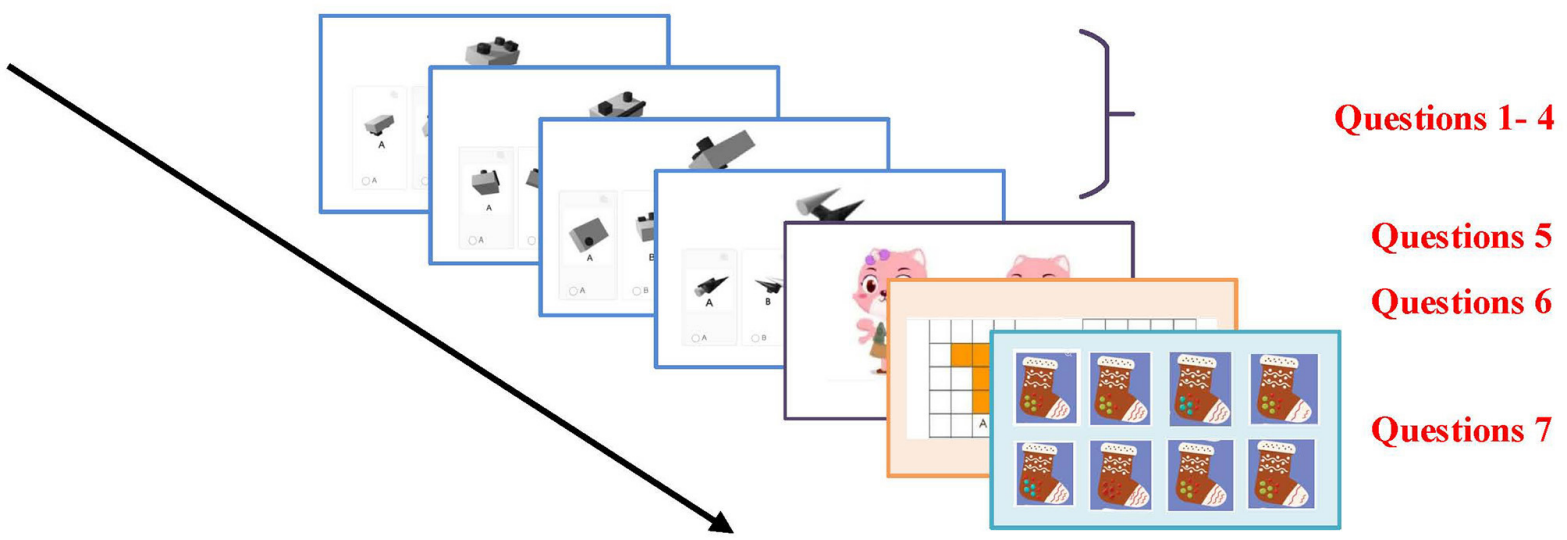
Schematic diagram of the visual-spatial skills testing task.

Visual-spatial skills are a key measure of the level of visual-spatial attention, reflecting the limits of a person's ability to process visual information in a given amount of time.This capacity indicator reflects the maximum capacity of the human visual system to perform effective processing of several visual stimuli while filtering out irrelevant distractions. We designed an online question-and-answer applet containing seven questions that comprehensively assessed the visual-spatial skills of the test subjects (the specific flow is shown in Figure 5).

These questions cover a variety of aspects such as spatial rotation ability, observation, visual sensitivity, perception of scale and size, and detail discrimination. First, we set up four graph rotation questions with different difficulty levels to suit testers of different ability levels. One specified figure will be displayed on the screen and five options will be provided, including a correct figure after rotation and four interference options. This type of question is primarily designed to test the examinees' ability to recognize and analyze spatial rotation. Question 5 shows two similar pictures and asks the examinees to identify the subtle differences between them. This type of question tests the examinees' observation and visual discrimination skills. In Question 6, the screen displays two flat shapes consisting of squares, and the examinees need to compare and determine the size of the areas of the two shapes. This question is designed to assess the examinees ability to perceive proportion and size. Question 7 shows eight pictures of similar objects that may differ in details. The examinees' task is to find a pair of identical pictures from among them. This question tests the examinees' ability to observe and recognize details. This test not only effectively assesses the overall ability of the examinees in the visual-spatial domain, but also helps to identify their strengths and weaknesses in specific visual tasks. After this quiz, the examinees were also required to perform a 6-point rating scale (1 = very easy, 2 = easy, 3 = average, 4 = somewhat difficult, 5 = difficult, and 6 = very difficult). The mean value of the difficulty rating was 2.97, which proves that the level of difficulty of the visual ability test material was appropriate. This experiment uses JavaScrip programming. At the same time, the correctness and reaction time of examinees are also recorded.

#### 4.2.2 Analysis of test results of visual-spatial skills

**Figure 6:**
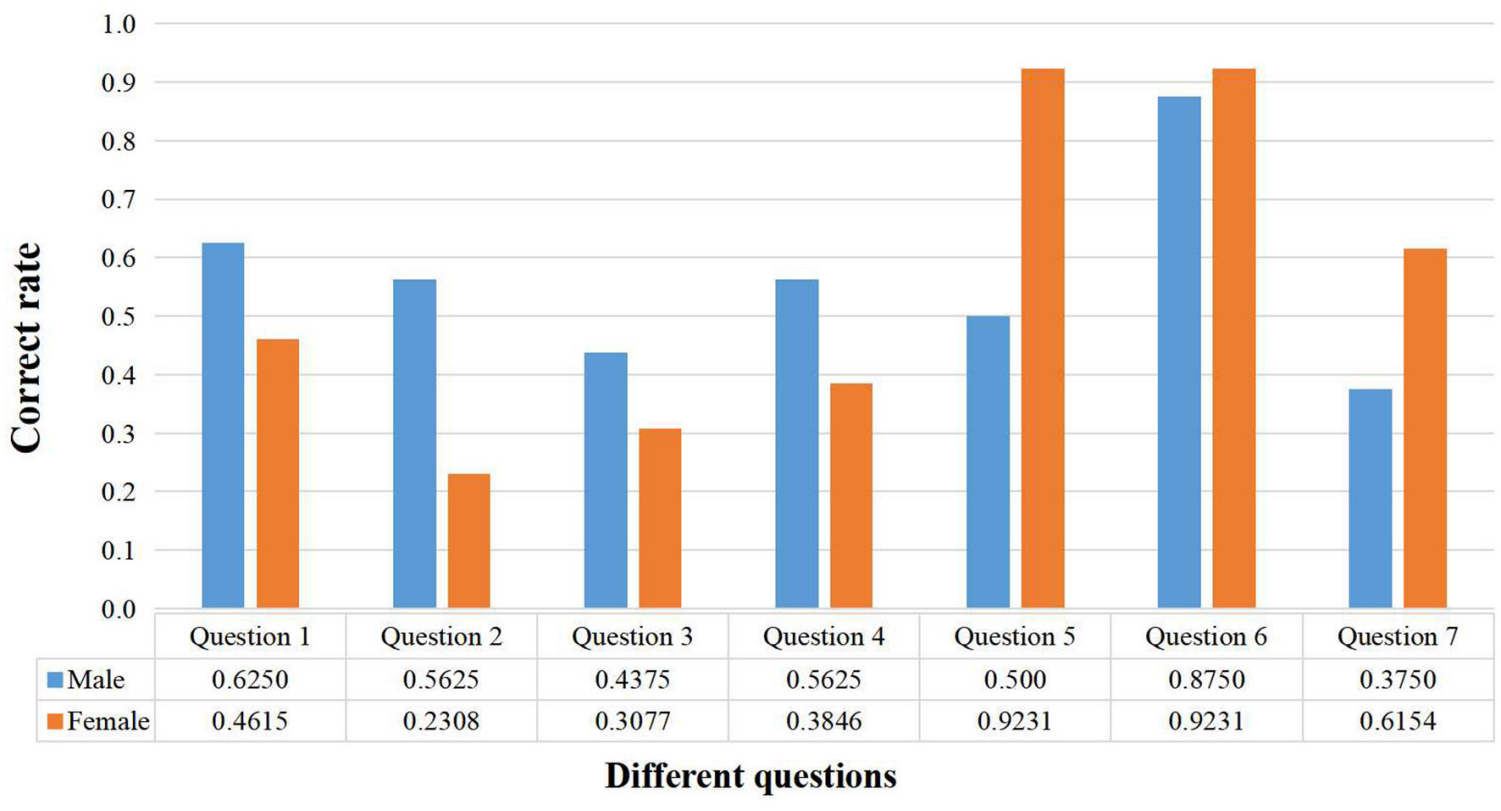
Results of visual-spatial skills test.

In this test, 55.17% of the participants were males and 44.83% were females, ensuring that the gender distribution was balanced within a certain range. The statistical results are shown in Figure 6.

In the first four questions involving spatial rotation ability, even though the difficulty of the questions varied, the overall performance of males was generally better than that of females. This may reflect differences between males and females in processing spatial information and problem solving, where males may have an advantage in dealing with these tasks.

The fifth question focuses on testing the examinees' observational and visual discrimination skills. It can be seen from the results that females scored significantly higher than males, indicating that females are better at observing and recognizing details.

In the sixth question, which tested proportionality and size perception, the performance of both sexes was very similar, indicating that gender differences were not significant on this type of perceptual task. In the seventh question, which tested the ability to recognize details, females performed significantly better than males, again emphasizing their strengths in dealing with more detailed and complex details. In terms of problem-solving time, the average time taken by females is 256.15 s, which is longer than that of males (220.75 s). This may reflect the fact that female students are more meticulous and conscientious in solving problems, which is one of the reasons why they have a higher rate of correctness on particular problems.

In addition, females' average rating of 3.46 for perceived test difficulty, compared to men's 2.56, suggests that they feel more challenged, which may enable them to pay more attention to detail and take each question more seriously. Taking these data together, although females' average scores were slightly higher, there was a clear difference between males' and females' performance on the different types of questions, revealing gender differences in visual-spatial skills.

### 4.3 Performance analysis of algorithms

In this paper, we compare the proposed deep neural network-based algorithm GNN-S with three traditional classification prediction methods, SVM (support vector machine), LR (logistic regression), and GaussionNB (gaussion naive bayes), as well as VAE-SVM (variational auto-encoders, support vector machine) and AE-SVM (auto-encoder, support vector machine) two deep learning based algorithms are compared and experimented to verify the effectiveness of our proposed method. Among them, the Auto-Encoder (AE) in AE-SVM consists of an encoder and a decoder. This method first inputs preprocessed data into the encoder and maps it to a low-dimensional latent space, then the decoder reconstructs the input data from the latent space and transforms the latent representations back to the original data space through a neural network layer. The AE then optimizes the parameters by comparing the difference between the minimized input data and the reconstructed data. Finally, the low-dimensional data representations extracted by the encoder are used as inputs and passed to the SVM to achieve the grade classification task. The self-encoder in VAE-SVM learns not only the latent representation of the data, but also its probability distribution. By introducing probabilistic and variational inference, the VAE can generate new data and capture the diversity and underlying structure of the data. This method also starts by feeding preprocessed data into the variational autocoder, after which the encoder maps the input data to a normal distribution with inputs of mean and variance. Next, the method extracts latent variables from the mean and variance of the encoder output and uses a reparameterization technique to decompose the random samples into deterministic operations and random noise components. The decoder then reconstructs the high-dimensional data from the sampled variables, optimizing the model parameters by minimizing the reconstruction error. Similarly, the final step of the algorithm is to take the latent variables output by the encoder as low-dimensional feature representations and input them into a SVM to achieve the achievement classification task.

The results of each experiment are the average values obtained by predicting and evaluating the training set (containing 60% of the links) and the test set (containing 40% of the links) formed by randomly dividing the original dataset for 100 times, and the experimental results are shown in Tables 3, 4. We have bolded the best results achieved by different algorithms in different evaluation metrics.

**Table 3:** Performance of different algorithms for performance classification prediction without considering visual-spatial skills.

**Methods**	**Accuracy**			**Precision**			**Recall**			**F1_Score**		
	**Male**	**Female**	**All**	**Male**	**Female**	**All**	**Male**	**Female**	**All**	**Male**	**Female**	**All**
SVM	0.8593	0.8270	0.8131	0.8872	0.8576	0.8288	0.8593	0.8270	0.8131	0.8678	0.8294	0.8172
LR	0.8460	0.7719	0.7892	0.8525	0.7732	0.7921	0.8460	0.7718	0.7892	0.7700	0.8451	0.7905
GaussianNB	0.6863	0.6882	0.7108	0.8085	0.6926	0.7443	0.6863	0.6882	0.7108	0.6955	0.6815	0.7039
VAE-SVM	0.5905	0.5586	0.5151	0.4545	0.4061	0.3713	0.5905	0.5586	0.5151	0.5061	0.4566	0.4156
AE-SVM	0.6169	0.4289	0.4529	0.4976	0.2966	0.3848	0.6169	0.4289	0.4529	0.5260	0.3062	0.3478
GNN-S	**0.9549**	**0.8774**	**0.9112**	**0.9546**	**0.8897**	**0.9146**	**0.9549**	**0.8774**	**0.9112**	**0.9504**	**0.8759**	**0.9081**

The bold values indicate the best results achieved by different algorithms in different evaluation metrics.

**Table 4:** Performance of different algorithms for performance classification prediction under considering visual-spatial skills.

**Methods**	**Accuracy**			**Precision**			**Recall**			**F1_Score**		
	**Male**	**Female**	**All**	**Male**	**Female**	**All**	**Male**	**Female**	**All**	**Male**	**Female**	**All**
SVM	0.8992	0.9468	0.9036	0.9552	0.9123	0.9042	0.9468	0.8992	0.9036	0.9494	0.9007	0.9035
LR	0.9125	0.8023	0.8182	0.9197	0.8177	0.8210	0.9125	0.8023	0.8183	0.9121	0.8065	0.8191
GaussianNB	0.7680	0.7890	0.7773	0.8418	0.8099	0.7950	0.7681	0.7890	0.7773	0.7725	0.7750	0.7756
VAE-SVM	0.6365	0.5989	0.5449	0.4908	0.4315	0.4114	0.6365	0.5989	0.5449	0.5486	0.4977	0.4459
AE-SVM	0.6234	0.6367	0.5902	0.5867	0.4996	0.5556	0.6367	0.6234	0.5902	0.5890	0.5565	0.5412
GNN-S	**0.9971**	**0.9705**	**0.9745**	**0.9972**	**0.9727**	**0.9747**	**0.9971**	**0.9965**	**0.9745**	**0.9972**	**0.9706**	**0.9744**

The bold values indicate the best results achieved by different algorithms in different evaluation metrics.

Table 3 shows the comparative performance results of different algorithms for predicting students' programming language exam scores by directly utilizing various data from students' online classes without considering visual-spatial skills. We can observe significant differences in classification prediction between different algorithms. Among them, LR, GaussianNB, VAE-SVM, AE-SVM algorithms are more uneven in classification prediction evaluation, especially in evaluating the dataset related to female students. Although SVM shows more balanced results in the performance metrics of classification prediction evaluation for male and female students, the GNN-S algorithm performs better. For the female student data, the GNN-S prediction results are clearly not as good as the classification prediction results for the male students, but overall the accuracy is very high in all the evaluation metrics, which shows the power of the algorithm on a specific subset of students.

As shown in Table 4, after considering the visual-spatial skills, we find that all algorithms have improved in all evaluation metrics. Among them, SVM continues to show stable and balanced performance in all performance indicators. LR, GaussianNB, VAE-SVM, AE-SVM also show significant improvement in all indicators after adding the attention mechanism, which indicates that visual-spatial skills help a lot in predicting students' grades. Among them, the LR algorithm shows the most obvious performance, which may be due to the fact that the LR algorithm can focus more on those features that contribute most to the prediction results.

This mechanism is particularly helpful in selecting key information for learning when the features of the dataset are diverse and complex, and substantially improves the prediction of student performance. The GNN-S algorithm proposed in this paper achieves almost near-perfect prediction performance on the male dataset and improved prediction performance on the female dataset with the addition of the attention mechanism, which may be due to the fact that the GNN-S algorithm is able to capture students' achievement strengths in visual-spatial skills more efficiently and translate such strengths into a key factor in improving the prediction of students' performance.

**Figure 7:**
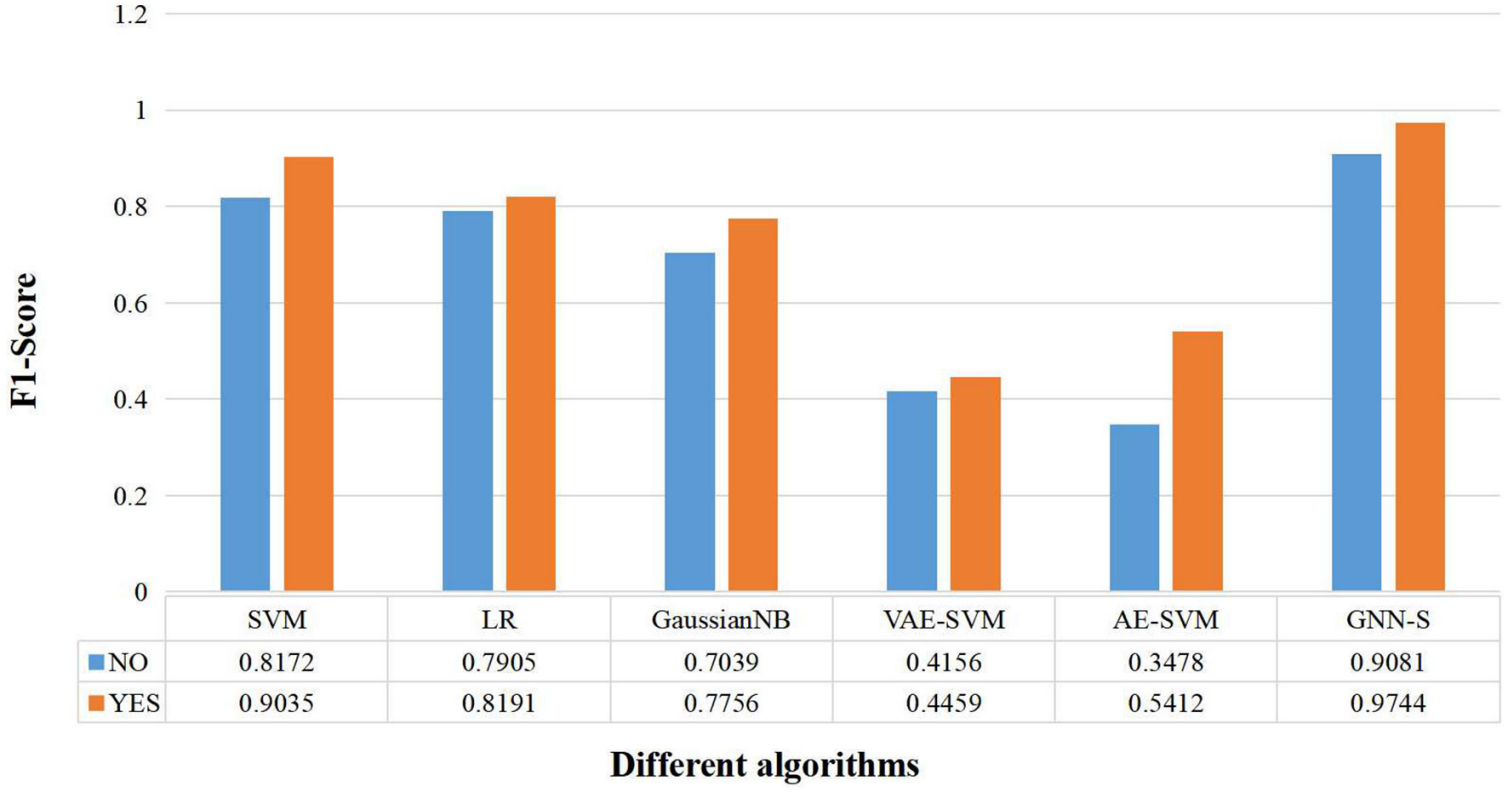
Performance comparison of different algorithms when predicting grades with or without considering visual-spatial skills.

Finally, we again compared the *F*1_*Score* results obtained by different algorithms with or without visual-spatial skills as an attribute feature for the algorithms' design (shown in Figure 7). We can see that, firstly, the algorithm GNN-S in this paper, regardless of whether the visual-spatial skills feature of the samples is considered or not, demonstrates the best performance among the algorithms. Secondly, the classification prediction results of each algorithm are improved after adding visual-spatial skills, e.g., AE-SVM, the accuracy is improved by 19.34%. This phenomenon fully illustrates the strong correlation between visual-spatial skills and students' programming performance. The incorporation of the visual-spatial skills mechanism provides a tool for the students' achievement categorization prediction algorithm to capture and process the differences between the achievements, which can effectively improve the accuracy of the algorithm.

## 5 Conclusions

This paper aims to explore the potential connection between visual-spatial skills and students' performance, analyze students' learning behaviors, learning outcomes and other learning-related factors through big data technology, and propose a model based on deep learning to predict students' performance. The model collects multidimensional data from students' online classrooms and adds data enhancement operations in the data preprocessing process, which enables the model to better learn the features and patterns of the data by introducing more data samples and diversity, thus improving the performance of the model on various tasks. The experimental results show that the method is greatly improved in accuracy compared to traditional prediction algorithms, and it is found that visual-spatial skills play an important role in the learning process of students' programming language courses. By collecting diversified students' data, this research can achieve automatic evaluation and prediction of students' learning effects, while providing teachers with timely feedback, establishing a personalized students' training model, and forming a closed-loop learning mechanism so as to improve students' motivation to learn. At the same time, this research also helps to improve the teaching efficiency of teachers in the classroom, making the teaching process more efficient and convenient, and realizing the personalization and refinement of teaching.

## Data availability statement

The raw data supporting the conclusions of this article will be made available by the authors, without undue reservation.

## Ethics statement

The studies involving humans were approved by Ethics Committee of Huaiyin Institute of Technology. The studies were conducted in accordance with the local legislation and institutional requirements. The participants provided their written informed consent to participate in this study.

## Author contributions

MJ: Conceptualization, Data curation, Methodology, Project administration, Supervision, Writing – original draft, Writing – review & editing. JL: Data curation, Methodology, Software, Validation, Visualization, Writing – original draft. BC: Conceptualization, Methodology, Project administration, Writing – original draft, Writing – review & editing. ZL: Validation, Visualization, Writing – original draft.
